# Clinical Remarks by Prof. J. F. Miner, M. D., on Surgical Cases Occurring in the Buffalo Hospital of the Sisters of Charity

**Published:** 1871-01

**Authors:** W. W. Miner


ART. VII.—*Clinical Remarks by* PROF. J. F. MINER, M. D., *on Surgical Cases occurring in the Buffalo Hospital of the Sisters of Charity.*

REPORTED BY W. W. MINER.

CASE XI.—*Hydrocele.*—Associated with hydrocele on the left side of the scrotum, this patient presents also scrotal hernia of long standing on the right. As to the pathology of hydrocele, you understand that the testicle, during fœtal life, descends from its position in the abdomen posterior to the peritoneum; and, in its descent into the scrotum, pushes down before it two layers of peritoneal tissue: of these layers, which are the tunica vaginalis the one nearest to and investing more or less the testicle, is the visceral layer; while, between this and the parietal layer, which is attached to the scrotum, an interval may exist, which is the cavity of the tunica vaginalis, and into which the fluid of hydrocele is effused. A variety called encysted hydrocele is known, in which the cysts have a peculiar areolar investment distinct from the tunica vaginalis; and the cavity formed is said to lead out from the spermatic tubes of the testis or epididymis, to which the cyst is closely adherent: this variety may be complicated with simple hydrocele of the cavity of the tunica vaginalis, or of the serous investment of the cord, between which, in treatment, no distinction is required.

On examining hydrocele with a light in a dark room, the translucency of the fluid accumulation will very often furnish you valuable evidence as to its character: this, with the smooth oval form of the swelling, its slow growth, and absence of pain, and of impulse on coughing, will render diagnosis clear.

Two methods of treatment are used in this affection—one of these effects temporary relief, and the other radical cure. By plunging a trocar into the cavity from in front, taking care not to wound the testicle, the fluid contents of the sac may easily be evacuated. This fluid is usually of a light straw color, and of varying quantity, sometimes amounting to twenty ounces: it is sometimes of dark color, from admixture with blood or other material: it generally reaccumulates after tapping, though sometimes it does not. In attempting the radical cure of hydrocele, setons drawn through the cavity of the sac have been in use: these may be attended with serious inflammation and extensive suppuration. Free exposure of the cavity of the sac by incision is sometimes made, allowing the gradual closure of the cavity by granulation: this procedure, also, is not unattended with danger. I believe it best to make use of an injection of iodine. After having removed the fluid contents of the sac by tapping, one or two drachms of tincture of iodine may be injected through the canula, and by manipulation every part of the sac be brought into contact with this irritating fluid. The object of this injection is not to cause extensive inflammatory conditions, but simply by stimulation to restore the natural harmony between the secretory and absorbent functions of the tunica vaginalis, or other serous structures from which hydrocele is derived, and from whose non-absorption this affection has origin.

Case XII.—*Transplantation.*—We now desire to make trial before you of a new method of operation very recently proposed by

M. Reverdin, an *interne* in one of the hospitals of Paris. The little boy brought before you received, three months since, a severe injury to his foot, causing considerable suppuration of the parts surrounding the os calcis and the ankle joint. Though the tissues have been nicely reproduced and present luxuriant granulations, the heel is altogether destitute of a covering of skin. The denuded surface is several square inches in extent, and is limited by skin of low vitality, and of little disposition of propagation. This condition promises to remain for an indefinite period.

Within the last few months it has been found in Paris, in the case of an ulcer following a severe burn, and which refused to heal, that bits of healthy skin, when removed with the lancet from a convenient part of the body, and carefully inserted upon the raw surface of the ulcer, formed living attachment there, becoming islets of healthy skin of an actively self-propagating character. In this manner the whole surface of the ulcer was soon furnished with an appropriate covering.

The skin consists, you are aware, of layers of epithelial cells, which have their origin in the *rete mucosum* close down upon the vascular, papillary surface of the corium; that each new layer, as it springs up upon this vascular network, displaces that which preceded it; and thus the successive desquamating layers of the skin have their origin. It seems that the cells of the *rete mucosum* need for their propagation only the advantages of vascular supply, and that they then proliferate readily. There seems, however, to be something of a limit to the extent of their proliferation, as in the case before you, the natural process of epithelial restoration seems to be stopped on the borders of the raw granulating surface of the heel. By simply implanting normal epithelial cells from the *rete mucosum*, within sufficiently near intervals from each other, upon this highly vascular granulating tissue, it is supposed that denuded surface may be provided to any extent with proper epithelial covering.

After inducing anæsthesia in the case in hand, four small bits of skin of the size of a kernel of rice were obtained from the leg, with a forceps and scissors. The depth of the incission was just enough so that a slight exudation of blood would appear from points of the surface beneath. These four pieces were then carefully placed upon the granulations, at intervals of about an inch from each other, being insinuated into slight incisions made in the granlulating surface. The bits thus implanted were at length held in place by adhesive straps.

At the end of a week, on the second appearance of the patient before the class, it was seen that each of the four pieces of transplanted skin was firmly attached to the surface of the foot, and that they were considerably increased in size; they appeared, at a little distance, somewhat like spots of sloughing material, though closer examination showed that these bits were in an active state of proliferation.

Seven weeks after the operation it was found that complete union of the epithelial growths had occurred, and that the portion of the denuded surface that had been furnished with germinal points was quite covered with newly formed epithelium. The remaining portion of the granulating surface was then implanted as in the former operation. A little resolution in the young patient obviated the use of any anæsthetic whatever at this time. This latter attempt promises equally good results with those already obtained, so that very soon the heel will be provided with a firm, thick and healthy covering of skin.

The benefits which probably are to be obtained from this method of operation, it can hardly be possible for those of you who have had no experience with sores, burns, and chronic ulcers, to sufficiently appreciate. I recall to mind a case which formerly occurred at this institution, in which the attending surgeon attempted, by an auto-plastic operation, to obtain integument from one leg for an old ulcer on the other leg. This operation, of course, required constraint for a considerable time, and was attended with much inconvenience, though the object in view could indeed thus be obtained. It is somewhat remarkable that each of our transplanted germs should have remained adherent, and maintained their growth. This is a better result than those who have written on the subject state to be generally obtained. The simplicity, efficiency and utility of this operation of transplantation, recommend it most strongly to general adoption by the surgical profession. If the operation is as generally practicable as it seems to be, it cannot but constitute a remarkable surgical achievement.

